# Inherited Type-1 renal tubular acidosis with short stature: a rare case report from Nepal

**DOI:** 10.1097/MS9.0000000000004080

**Published:** 2025-10-13

**Authors:** Dipesh Kumar Singh, Avinas Rai, Robin Maskey

**Affiliations:** Department of Internal Medicine, B.P. Koirala Institute of Health Sciences, Dharan, Nepal

**Keywords:** alkali treatment, molecular genetic test, nephrocalcinosis, rare disease, Type-1 distal RTA (dRTA)

## Abstract

**Introduction:**

Distal renal tubular acidosis (dRTA), or Type-1 RTA, is a rare disorder characterized by the failure of alpha-intercalated cells in the distal nephron to excrete hydrogen ions. This leads to a normal anion gap metabolic acidosis, hypokalemia, hypercalciuria, and persistently alkaline urine. Complications include nephrocalcinosis, growth retardation, and skeletal abnormalities. Hereditary forms, often involving mutations in genes like SLC4A4, typically present in early life and may include systemic features such as sensorineural hearing loss. The present case indeed reports and summarizes both clinical symptoms and diagnosis, long-term outcomes, genetic inheritance, epidemiology, and current treatment options, with the aim of shedding more light onto this rare disorder, with a specific focus on the diagnostic challenges posed by a delayed presentation and the importance of genetic evaluation.

**Case Presentation:**

We report a 27-year-old female with progressive lower limb weakness and chronic muscle cramps. She had a longstanding history of renal calculi since adolescence and marked growth delay. Laboratory tests revealed normal anion gap metabolic acidosis, urinary pH >7.0, hypokalemia, hypophosphatemia, and vitamin D deficiency. Imaging showed bilateral nephrocalcinosis. Genetic testing identified a pathogenic SLC4A4 mutation, confirming inherited dRTA. Treatment with potassium citrate, sodium bicarbonate, vitamin D, calcium, and iron supplementation led to significant clinical improvement.

**Discussion:**

This case highlights the diagnostic challenges of hereditary dRTA and its systemic burden. Nephrolithiasis, short stature, and persistent metabolic acidosis should prompt consideration of RTA. Although SLC4A4 is commonly linked with proximal defects, its mutation can present with distal tubular dysfunction, emphasizing the role of genetic analysis.

**Conclusion:**

Hereditary dRTA is a rare but treatable condition. Early diagnosis and lifelong alkali therapy can significantly reduce complications, including chronic kidney disease and skeletal deformities. This case reinforces the need for heightened awareness among clinicians to consider dRTA in patients presenting with chronic fatigue, muscle weakness, short stature, and recurrent nephrolithiasis. Furthermore, it underscores the value of genetic evaluation in uncovering the precise etiology, allowing for tailored management and genetic counseling.

## Introduction

Renal tubular acidosis (RTA) represents a group of heterogeneous disorders characterized by the kidney’s inability to maintain systemic acid–base homeostasis, leading to a state of chronic, normal anion gap (hyperchloremic) metabolic acidosis. This occurs due to impaired hydrogen ion (H^+^) excretion or bicarbonate (HCO_3_−) reabsorption within various segments of the renal tubule. Among the subtypes, the current case focus is placed on the most common type of RTA, Type-1 RTA or Distal RTA (dRTA), which is a rare chronic genetic disorder characterized by an inability of the alpha-intercalated cells of distal nephron to secrete hydrogen ions in the presence of metabolic acidosis[1]. This defect hampers the kidney’s ability to acidify urine despite systemic acidosis, resulting in persistently high urinary pH (typically >5.5), hypocitraturia, and often hypercalciuria. These abnormalities contribute to nephrocalcinosis and calcium phosphate stone formation[2]. Clinically, the condition manifests as failure to thrive, polyuria, hypokalemia-induced muscle weakness, rickets in children, and osteomalacia in adults. In some hereditary forms, particularly autosomal recessive dRTA, sensorineural hearing loss may also occur, further compounding the clinical burden.

**HIGHLIGHTS**

- Hereditary distal renal tubular acidosis is often underdiagnosed and presents early in life.
- Persistent metabolic acidosis with high urinary pH and hypokalemia are key diagnostic clues.
- Genetic testing can help differentiate subtypes and guide family counseling.
- Timely alkali therapy and potassium supplementation can reverse systemic symptoms.
- Early diagnosis prevents complications like nephrocalcinosis, growth failure, and CKD.

The etiology of dRTA is multifactorial. In pediatric populations, the primary form is frequently due to genetic mutations affecting components of the acidification machinery, such as ATP6V1B1, ATP6V0A4, and SLC4A1. These inherited forms may present with severe metabolic acidosis, hypokalemia, rickets, growth retardation, and sensorineural hearing loss. Conversely, adult-onset dRTA is more commonly secondary to autoimmune diseases such as Sjögren’s syndrome and systemic lupus erythematosus, chronic obstructive uropathies, and exposure to nephrotoxic drugs like amphotericin B and ifosfamide[3].

The diagnosis of dRTA requires a high index of clinical suspicion. Hallmark features include persistent metabolic acidosis with a normal anion gap, hypokalemia, nephrocalcinosis, and calcium phosphate nephrolithiasis. A urinary pH persistently above 5.5 in the context of systemic acidosis is a key diagnostic clue. The definitive diagnosis can be confirmed with an acid-loading test using oral ammonium chloride; failure to lower urinary pH below 5.3 supports the diagnosis. Incomplete dRTA, a forme fruste variant, lacks overt metabolic acidosis and is often unmasked only through such provocative testing. The pathophysiological triad of dRTA:alkaline urine, hypocitraturia, and often hypercalciuria, which predisposes patients to recurrent calcium phosphate stones and nephrocalcinosis[4]. Additionally, persistent hypokalemia may contribute to muscle weakness and cardiac dysrhythmias.

Recent advances in molecular diagnostics have facilitated the early identification of inherited dRTA. Genetic testing enables the detection of specific mutations, which is particularly valuable for family counseling and in cases of early-onset disease. Nonetheless, the utility of genotyping in acquired or adult cases remains a subject of ongoing research. Management of dRTA centers on alkali replacement therapy, most commonly using potassium citrate. This corrects acidosis, replenishes potassium, inhibits stone formation, and helps preserve bone mineral density[5]. In cases with severe hypokalemia, potassium supplementation may be prioritized. Lifelong treatment is often necessary, and periodic monitoring is essential to assess response and prevent complications.

Importantly, dRTA is now recognized as a chronic condition with potential long-term consequences, including chronic kidney disease, skeletal deformities, and growth failure. The present case indeed reports and summarizes both clinical symptoms and diagnosis, long-term outcomes, genetic inheritance, epidemiology, and current treatment options, with the aim of shedding more light onto this rare disorder. Being a chronic condition, dRTA also deserves attention in the transition between pediatric and adult nephrology care. The present case indeed reports and summarizes both clinical symptoms and diagnosis, long-term outcomes, genetic inheritance, epidemiology, and current treatment options, with the aim of shedding more light onto this rare disorder, with a specific focus on the diagnostic challenges posed by a delayed presentation and the importance of genetic evaluation.

## Methods

Study design: The study is a case report (prospective, unicenter, formal, consecutive, and clinical). The work has been reported in line with the CARE 2013 criteria[6]. The article is compliant with the TITAN guidelines 2025[7].

## Case presentation

A 27-year-old female presented to the Internal Medicine OPD of a tertiary care center with chief complaints of generalized weakness of bilateral lower limbs accompanied by muscle cramps. The patient said that she was apparently well until the age of 12 years, when she first began experiencing right-sided lumbar pain. The pain was rated 7/10 on the numeric pain scale, compressive in nature, radiating to the back, aggravated by physical exertion, and partially relieved by rest. She was diagnosed with right renal calculi at that time and had undergone Ayurvedic treatment for approximately 2 years. During this period, she frequently sought symptomatic relief using analgesics from local polyclinics.

Approximately 1.5 years back to her current presentation, she experienced intermittent episodes of flank pain, often associated with dysuria. She developed a new onset of lower limb weakness and was admitted for evaluation. Imaging studies at that time revealed left-sided nephrolithiasis, which was surgically treated. And laboratory findings at that time showed hypokalemia, for which she received supportive therapy.

Five days back prior to admission, the patient had weakness of both lower limbs, accompanied by generalized muscle cramps that were exacerbated by activity and relieved with rest. She also had chronic fatigue, decreased appetite, and a progressive decline in her daily functional activities. Despite maintaining a normal diet, she was consistently being the shortest among her peers since early childhood.

There was no history of polydipsia, polyuria, urinary tract infections, hematuria, or heat intolerance. She denied any history of consanguinity or familial occurrence of renal disease, metabolic disorders, or growth abnormalities. No ocular abnormalities were noted.

On general examination, the patient was alert, oriented, and hemodynamically stable with a pulse of 90 bpm, blood pressure of 120/80 mmHg, respiratory rate of 20 breaths per minute, temperature of 98°F, and SpO_3_ of 99% on room air. Anthropometric measurements revealed a height of 132 cm, weight of 40 kg, BMI of 22.9 kg/m^2^, arm span of 139 cm, upper segment length of 70 cm, and lower segment length of 62 cm, confirming short stature.

Systemic examination was unremarkable. Cardiovascular and respiratory systems were within normal limits, with normal heart sounds (S1, S2), equal chest expansion, and no deformities. Abdominal examination revealed a soft, non-tender abdomen without organomegaly. Neurological examination revealed no focal deficits.

**Figure 1.**
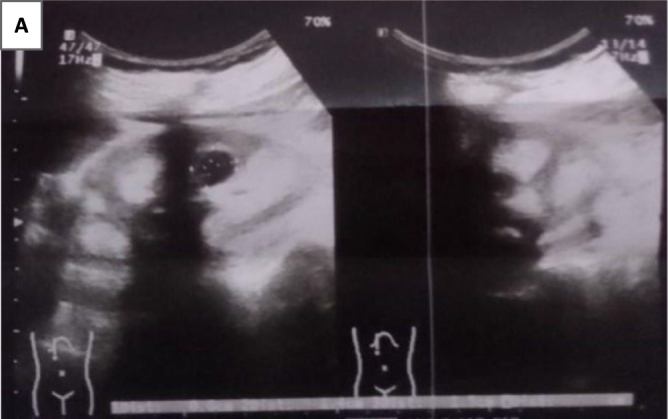
The ultrasonography findings of this patient revealed bilateral nephrolithiasis (multiple) and bilateral nephrocalcinosis.

Initial laboratory investigations demonstrated a metabolic acidosis with a blood pH of 7.25 and a serum bicarbonate level of 13 mmol/L. Electrolyte analysis revealed hypokalemia (2.80 mmol/L), hypocalcemia, and hypophosphatemia. Urine pH was measured at 7.0, indicating impaired distal acidification. Serum 25-hydroxyvitamin D was deficient. Renal ultrasonography revealed bilateral nephrocalcinosis with evidence of past renal calculi. Complete blood count showed microcytic hypochromic anemia suggestive of iron deficiency. Liver function tests were within normal limits. Serological tests for HIV, HBsAg, and HCV were nonreactive. Figure 1 shows the picture of her ultrasonography findings revealed bilateral nephrolithiasis (multiple) and bilateral nephrocalcinosis.

The examination and investigations including non-anion gap metabolic acidosis, persistently high urinary pH, hypokalemia, growth retardation, nephrocalcinosis, and vitamin D deficiency – was highly suggestive of Type-1 (distal) RTA. Given the chronicity of symptoms and early onset of growth failure, a genetic etiology was considered. Subsequent genetic testing confirmed a mutation in the SLC4A4 gene, consistent with inherited dRTA. Biochemical investigations are shown in Table 1.

**Table 1:** Biochemical parameters of the patient

Investigations	Results	Reference range value
Renal Function		
Urea (mg/dL)	29.35	10–50
Creatinine (mg/dL)	0.86	0.3–1.2
Random glucose (mg/dL)	113.75	<140
Serum electrolytes		
Sodium (700 AU) (mmol/L)	139.70	135–146
Potassium (700 AU) (mmol/L)	**2.8**	3.5–5.0
Thyroid function		
FT3 (pg/mL)	2.52	1.21–4.18
FT4 (pg/mL)	14	8.9–17.2
TSH (IU/mL)	4.34	0.3–4.5
Anti-TPO (IU/mL)	2.89	<30
Urine analysis		
Urine protein	Negative	
Urine epithelial cell (HPF)	**2–4**	
Urine RBC (HPF)	Not seen	
Urine WBC (HPF)	**0–2**	
Urine sugar	Negative	
Acid–base		
Arterial blood pH	**7.25**	7.35–7.45
PO_2_ (mm Hg)	**99**	80–105
PCO_2_ (mm Hg)	**30.1**	35–45
HCO_3_ (mmol/L)	**13**	22–28
Electrolytes		
Na^+^ (mmol/L)	**146**	138–146
K^+^ (mmol/L)	**3.1**	3.5–4.9
Hormones and vitamins		
Vitamin B12 (pg/mL)	483	200–1100
Vitamin D (ng/mL)	**11.8**	30–100
Renin (µIU/mL)	41.8	4.2–45.6
Aldosterone	293	70–300
Total calcium (Beckman) (mg/dL)	**8.0**	8.4–10.2
Others		
Urine PH	**7.0**	<5.5
Uric acid (Beckman) (mg/dL)	3.12	1.5–6.0
Magnesium (700 AU) (mmol/L)	1.07	0.66–1.07
Ionized calcium (mmol/L)	**1.04**	1.05–1.27
Phosphorus (700 AU) (mg/dL)	**1.48**	2.0–5.1
Antinuclear antibody (ANA) (AU/mL)	11.0	<40
PTH (parathormone) (pg/mL)	32.9	6–80
Cortisol (ng/mL)	99.1	72.6–322.8

**Figure 2.**
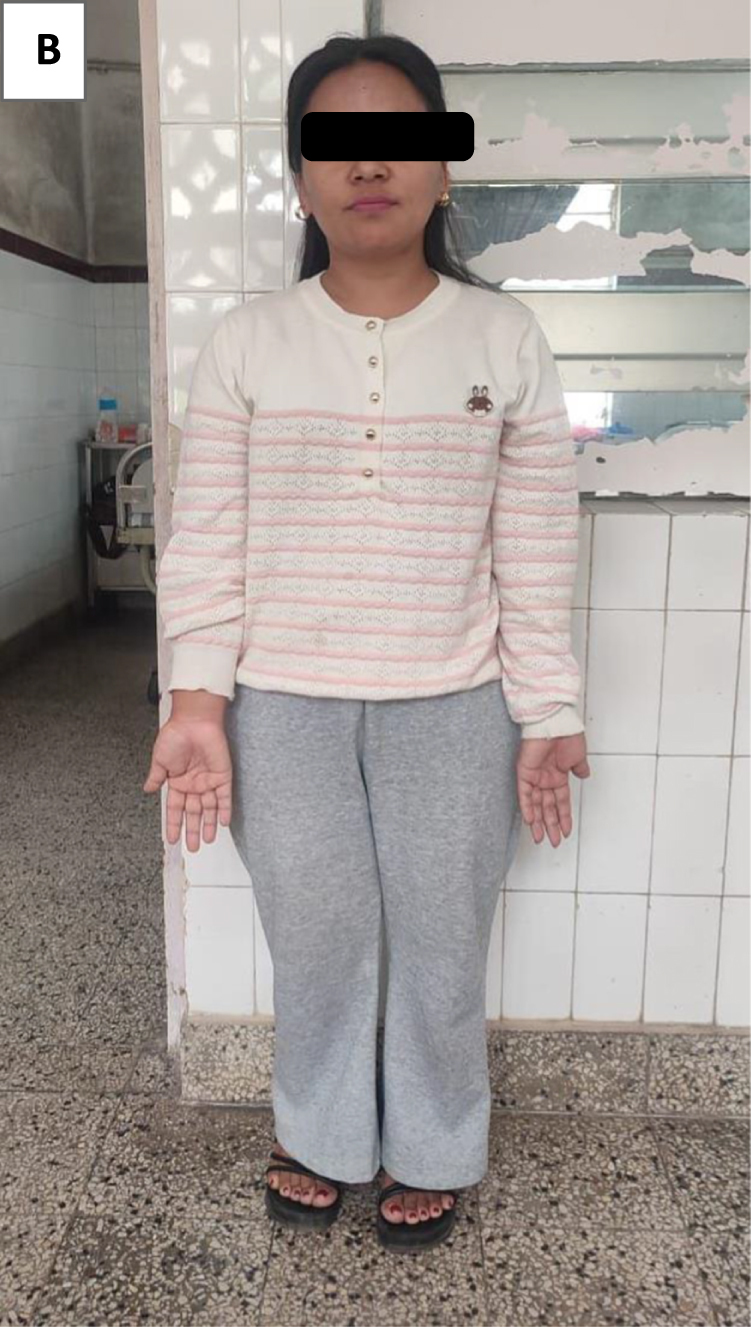
Picture of this patient on presentation, showing her short stature.

The patient was managed with intravenous hydration, potassium chloride supplementation, oral sodium bicarbonate for correction of acidosis, vitamin D supplementation, and oral iron therapy. Her clinical symptoms, including limb weakness and cramps, improved significantly during the hospital stay. She was discharged in a stable condition on Sodium bicarbonate tablet, oral potassium citrate syrup, vitamin D supplements, and calcium tablet. She was advised to follow up in the Medicine outpatient department after 3 months or earlier if symptoms recurred. After 3 months of follow-up, she is symptomatically better, her lab parameters are within normal limits. A picture of this patient during presentation time is shown in Figure 2.

## Discussion

dRTA, or Type-1 RTA, results from the failure of the distal nephron to adequately secrete hydrogen ions, leading to systemic non-anion gap metabolic acidosis with a persistently alkaline urine (pH > 5.5). This defect also promotes hypercalciuria and hypocitraturia, creating a milieu favoring calcium-phosphate stone formation and progressive nephrocalcinosis. This case exemplifies a classic presentation of hereditary dRTA, with a unique constellation of findings including growth retardation, recurrent nephrolithiasis, and hypokalemia – hallmark features of the disorder[8].

The patient’s long history of lumbar pain and recurrent renal calculi, beginning as early as 12 years of age and initially managed with nonconventional therapy, culminated in bilateral nephrocalcinosis and reflects the characteristic pathophysiology of hereditary dRTA. Bone buffering of the chronic acid load increases urinary calcium excretion, while hypocitraturia limits calcium complexation, together with the persistently alkaline urine, creating an environment that favors calcium-phosphate precipitation and intrarenal calcification. This delayed definitive diagnosis – common in hereditary dRTA – contributed to long-term complications such as nephrocalcinosis and growth failure. Clinicians should therefore consider dRTA in any patient presenting with recurrent nephrolithiasis, short stature, and persistent non-anion gap metabolic acidosis.

Her acute presentation with muscle cramps and bilateral limb weakness corresponded to profound hypokalemia. Failure of distal hydrogen secretion raises luminal electronegativity and, together with secondary hyperaldosteronism, augments distal sodium reabsorption and potassium secretion, causing renal potassium wasting. Chronic fatigue and poor appetite likely stemmed from this combined metabolic acidosis and potassium deficit.

Anthropometric measurements revealing marked short stature (height 132 cm with arm span greater than height) underscore the impact of chronic acidosis on skeletal growth. Acid retention stimulates bone mineral dissolution to buffer hydrogen ions, mobilizing calcium and phosphate and impairing bone mineralization, while concurrent vitamin D deficiency further limits calcium absorption. Similar growth impairment and rickets-like changes are well documented in pediatric dRTA when acidosis is untreated.

Laboratory findings of non-anion gap metabolic acidosis, hypokalemia, hypophosphatemia, and an inappropriately high urine pH confirmed distal acidification failure, while the absence of autoimmune markers excluded acquired causes. Ultimately, genetic testing revealed an SLC4A4 mutation, which is notably more often associated with proximal RTA and Fanconi syndrome but known in certain variants to produce overlapping or mixed tubular phenotypes^[9,10]^. This underlines the clinical heterogeneity of hereditary RTA and the importance of molecular diagnostics for precise classification. Although not detected here, ATP6V1B1 mutations are a classic cause of autosomal-recessive dRTA, frequently accompanied by early-onset sensorineural hearing loss, and remain important in differential diagnosis and family counseling.

Therapeutically, the patient’s management followed established guidelines. Alkali therapy using oral sodium bicarbonate corrected the acidosis, while potassium and vitamin D supplementation addressed electrolyte and bone metabolism issues[11]. Her positive response to inpatient therapy with improved muscular strength and symptom resolution mirrors outcomes in other documented cases where timely correction of acidosis mitigates systemic effects. She was discharged on potassium chloride, which serves the dual purpose of replenishing potassium and preventing stone formation.

In sum, this case highlights the need for early recognition of hereditary dRTA in patients with recurrent nephrolithiasis, unexplained growth failure, or persistent non-anion gap metabolic acidosis. Linking these clinical cues to timely molecular testing (including SLC4A4 and ATP6V1B1) enables precise diagnosis, targeted counseling, and early alkali therapy, which collectively preserve renal function, reduce stone burden, and support normal growth and musculoskeletal health. Compared to published cases, this patient underscores the importance of early clinical suspicion, especially in those with a history of recurrent kidney stones and unexplained growth delay. The long delay from symptom onset to diagnosis in this case highlights a significant knowledge gap in recognizing and investigating hereditary dRTA early, particularly in resource-limited settings.

## Conclusion

This case presents a detailed and instructive example of hereditary dRTA in a 27-year-old female with growth retardation, bilateral nephrocalcinosis, persistent hypokalemia, and non-anion gap metabolic acidosis. Her history of recurrent renal calculi since adolescence, progressive limb weakness, and eventual confirmation of SLC4A4 mutation illustrate the chronic and often under-recognized nature of hereditary RTA.

Despite the late diagnosis, the patient responded well to standard treatment with alkali and potassium supplementation, highlighting the reversible nature of symptoms when appropriately managed. This case reinforces the need for heightened awareness among clinicians to consider dRTA in patients presenting with chronic fatigue, muscle weakness, short stature, and recurrent nephrolithiasis. Furthermore, it underscores the value of genetic evaluation in uncovering the precise etiology, allowing for tailored management and genetic counseling.

Early diagnosis and sustained medical therapy are crucial in preventing irreversible complications such as chronic kidney disease and bone deformities. As demonstrated here, timely intervention even in adulthood can significantly enhance patient outcomes and quality of life. This case reinforces the need for heightened awareness among clinicians to consider dRTA in patients presenting with chronic fatigue, muscle weakness, short stature, and recurrent nephrolithiasis. Furthermore, it underscores the value of genetic evaluation in uncovering the precise etiology, allowing for tailored management and genetic counseling.

## Data Availability

The data that support the findings of this study are available from the corresponding author upon reasonable request.

## References

[1] Giglio S Montini G Trepiccione F. Distal renal tubular acidosis: a systematic approach from diagnosis to treatment. J Nephrol 2021;34:2073–83. 33770395 10.1007/s40620-021-01032-y PMC8610947

[2] Magni G Unwin RJ Moochhala SH. Renal tubular acidosis (RTA) and kidney stones: diagnosis and management. Arch Esp Urol 2021;74:123–28. 33459628

[3] Mohebbi N Wagner CA. Pathophysiology, diagnosis and treatment of inherited distal renal tubular acidosis. J Nephrol 2018;31:511–22. 28994037 10.1007/s40620-017-0447-1

[4] Fuster DG Moe OW. Incomplete distal renal tubular acidosis and kidney stones. Adv Chronic Kidney Dis 2018;25:366–74. 30139463 10.1053/j.ackd.2018.05.007 PMC7932558

[5] Vallés PG Batlle D. Hypokalemic distal renal tubular acidosis. Adv Chronic Kidney Dis 2018;25:303–20. 30139458 10.1053/j.ackd.2018.05.003

[6] Riley DS Barber MS Kienle GS. CARE guidelines for case reports: explanation and elaboration document. J Clin Epidemiol 2017;89:218–35. 28529185 10.1016/j.jclinepi.2017.04.026

[7] Agha RA Mathew G Rashid R. TITAN Group. Transparency in the reporting of Artificial INtelligence – the TITAN guideline. Prem J Sci 2025;10:100082.

[8] Trepiccione F Walsh SB Ariceta G. Distal renal tubular acidosis: eRKNet/ESPN clinical practice points. Nephrol Dial Transplant 2021;36:1585–96. 33914889 10.1093/ndt/gfab171

[9] Wagner CA Unwin R Lopez-Garcia SC. The pathophysiology of distal renal tubular acidosis. Nat Rev Nephrol 2023;19:384–400. 37016093 10.1038/s41581-023-00699-9

[10] Liu J Shen Q Li G. Clinical and genetic analysis of distal renal tubular acidosis in three Chinese children. Ren Fail 2018;40:520–26. 30230413 10.1080/0886022X.2018.1487858 PMC6147104

[11] Soares SBM de Menezes Silva LAW de Carvalho Mrad FC. Distal renal tubular acidosis: genetic causes and management. World J Pediatr 2019;15:422–31. 31079338 10.1007/s12519-019-00260-4

